# Supplementary material for: Genetic polymorphisms in the serotonin, dopamine and opioid pathways influence social attention in rhesus macaques (Macaca mulatta)
Source: PLoS One. 2023 Aug 2;18(8):e0288108. doi: 10.1371/journal.pone.0288108 (PMC10395878; doi:10.1371/journal.pone.0288108)
Supplement: Attachment — Submitted filename: Plos1_Review_Report_Authors.pdf [file pone.0288108.s002.pdf]

In this article, the authors have investigated the effect of polymorphism at nine loci in eight genes on two measures of social attention and eight measures of behavioural patterns for primates. The data was collected on 109 rhesus macaques. The results supported previous findings as well as demonstrated a statistically significant effect of some of the previously unexplored polymorphism on social attention and behaviour pattern of rhesus macaques. The article is well-written and contributes significantly to the existing literature on the effect of genetic polymorphism on the negative emotion and social behaviour of primates. Below are some major and minor comments.

The name of the procedure implemented in this article is Bayesian MCMCglmm and it can mislead the reader. The author should use some alternative standard name for their procedure, for example, MCMC-based Bayesian GLMM. Note that the R package which implements MCMC based Bayesian GLMM method indeed is named “MCMCglmm”. However, the method should not be referred to as MCMCglmm.

The abbreviation NHP (line 229) is used without clarification. Please clarify all the abbreviations carefully and introduce them in the right place in the article. Also, use abbreviations wherever possible.

Use abbreviation instead of non-human primates at line 249.

As the same data is used repeatedly to test the significance of genetic polymorphisms on different response variables using different models, so an adjusted p-value in place of an ordinary p-value should be reported. In that case, however, it seems that some of the significant discoveries would turn out to be false discoveries. This is a major concern and must be addressed carefully.

The discussion section is too long. It can be shortened by discarding lines like “When treated with mirtazapine, a noradrenergic and specific serotonergic antidepressant, individuals with the STin 12 allele showed a significant reduction in their Hamilton Depression Rating Scale score compared to individuals with the low expressing STin allele. Mirtazapine has also been used in the treatment of social anxiety [149] and social phobia [150].”

Tuning MCMC is a challenging problem and improper selection of burn-in may lead to the incorrect posterior distribution, which in turn may incorrectly interpret a non-significant covariate to be significant. It would be better if the authors demonstrate some of the MCMC trace plots and posterior density as supplementary figures.

That said I think the article is novel and after addressing the above-mentioned concerns it can be accepted for publication.
